# Cost-Effectiveness of Differentiated Service Delivery for HIV Treatment: A Combined Mathematical Modeling Study of Four African Settings

**DOI:** 10.1093/ofid/ofag257

**Published:** 2026-06-19

**Authors:** Shiying You, Hae-Young Kim, Andrew N Phillips, Daniel T Citron, David Kaftan, Ingrida Platais, Loveleen Bansi-Matharu, Valentina Cambiano, Brooke E Nichols, Youngji Jo, Ronald S Braithwaite, Edinah Mudimu, Anna Bershteyn

**Affiliations:** Department of Population Health, NYU Grossman School of Medicine, NewYork, New York, USA; Department of Population Health, NYU Grossman School of Medicine, NewYork, New York, USA; Institute for Global Health, University College London, London, UK; Department of Population Health, NYU Grossman School of Medicine, NewYork, New York, USA; Department of Population Health, NYU Grossman School of Medicine, NewYork, New York, USA; Department of Population Health, NYU Grossman School of Medicine, NewYork, New York, USA; Institute for Global Health, University College London, London, UK; Institute for Global Health, University College London, London, UK; Department of Global Health, Boston University School of Public Health, Boston, Massachusetts, USA; Department of Public Health Sciences, School of Medicine, University of Connecticut, Farmington, Connecticut, USA; Department of Population Health, NYU Grossman School of Medicine, NewYork, New York, USA; Department of Decision Sciences, University of South Africa, Pretoria, South Africa; Department of Population Health, NYU Grossman School of Medicine, NewYork, New York, USA

**Keywords:** antiretroviral therapy, cost-effectiveness analysis, differentiated service delivery, HIV/AIDS, mathematical model

## Abstract

**Background:**

Differentiated service delivery (DSD) is increasingly available for HIV treatment. DSD has been shown to improve treatment retention, but DSD modalities incur higher costs than the clinic-based standard-of-care (SoC). We conducted a cost-effectiveness (CE) analysis to assess what DSD modalities, in what settings, would constitute an efficient use of limited HIV program resources.

**Methods:**

We adapted two validated mathematical models (*EMOD-HIV* and *HIV Synthesis*) to project HIV trends (incidence, prevalence, mortality), disability-adjusted life years (DALYs), and costs (2021 USD) arising from DSD versus SoC over 2022–2062 in four settings: South Africa, Malawi, Zambia, and a collective representation of African low- and middle-income countries (LMICs). We compared three DSD modalities: healthcare worker-managed community adherence groups (CAG), client-managed urban adherence group (UAG), and home ART delivery (HomeART). We calculated incremental cost-effectiveness ratios (ICERs) of DSD versus SoC from the health system perspective using country-specific CE thresholds, and performed one-way sensitivity analyses for key assumptions.

**Results:**

Community adherence groups (ICER: $274–$604/DALY averted) and UAG (ICER: $590–$720/DALY averted) were cost-effective for all country/model settings. HomeART was dominated by UAG in all settings. In nearly all settings, CE estimates of CAG were robust to uncertainty in DSD effectiveness (except Zambia), DSD costs, CE threshold (except South Africa), HIV-associated disability weights, and discount rates. Cost-effectiveness of UAG was highly sensitive to uncertainty in DSD effectiveness in all settings.

**Conclusions:**

Community adherence groups and UAG can provide cost-effective alternatives to the clinic-based SoC in multiple African settings.

## Teaser

Some HIV differentiated service delivery models cost more than clinic-based care, making cost-effectiveness essential to determine their value. Using two mathematical models in four African settings, we found that community and urban adherence groups can cost-effectively expand clinic-based care, while home-based care would be less cost-effective. These findings provide critical evidence to guide HIV resource allocation and policy decisions for scaling HIV treatment delivery.

Two-thirds of people living with HIV (PLHIV) reside in Africa, where HIV continues to be a leading cause of death [1]. Availability of antiretroviral therapy (ART) has reduced but not eliminated HIV as a major public health issue [2]. People living with HIV who remain adherent to ART for life can experience life expectancies similar to those of HIV-negative populations and can bring HIV viral loads to levels that are undetectable and untransmittable [3]. However, many PLHIV face challenges in remaining engaged in care for life, including logistical and psychosocial barriers [4]. Evidence suggests that ART-experienced patients who become lost to follow-up (LTFU) contribute to a growing proportion of advanced HIV, HIV-related deaths, and new HIV infections [5–7]. Concurrently, HIV care systems are treating larger volumes of PLHIV with stagnant budgets, which can lead to challenges in maintaining service quality, such as crowding and long wait times at care facilities [8]. These systems-level challenges can further exacerbate LTFU [4, 9].

Differentiated service delivery (DSD) has been developed as a person-centered care strategy to enhance ART retention and manage patient volumes [10]. DSD aims to tailor ART delivery based on individual needs while decongesting overburdened clinics and increasing the quality of care [11]. DSD modalities differ from the conventional standard-of-care (SoC) in terms of the cadre of healthcare workers involved, the location of the services provided, and the frequency of patient-provider interactions [8]. Four general types of DSD modalities have been studied: group-based client-managed DSD, group-based healthcare worker-managed DSD, individual facility-based DSD, and individual community-based DSD [12].

Evidence from existing studies indicates that DSD effectiveness is either equivalent or superior to SoC in a range of African settings [8, 10]. DSD offers comparable viral load suppression rates and improves ART retention compared with SoC [13]. However, although studies initially hypothesized that DSD would reduce ART delivery costs, several studies have revealed that the annual per-patient treatment cost is higher for most DSD modalities compared with SoC, particularly as SoC has become more efficient over decades of implementation [8, 11, 14]. The first “head-to-head” cost comparison of DSD to SoC was undertaken in Zambia and found that all four DSD modalities evaluated had higher annual per-patient costs compared with the SoC from the health system perspective [15].

In situations where a healthcare option has superior outcomes but increased cost, cost-effectiveness analysis (CEA) can be informative to determine whether investing in the service would be an efficient use of limited resources. CEA is particularly important in settings with severe healthcare budget constraints, as is the case in Zambia and other countries in the region. This study aimed to evaluate the cost-effectiveness (CE) of DSD modalities, leveraging existing data on the costs and effectiveness of DSD and SoC. We analyzed epidemiological and cost forecasts for three DSD modalities in four African settings using two previously validated mathematical models over 2022–2062. This research was undertaken to support decision-making regarding DSD scale-up in Africa, enhance the understanding of the long-term health and economic implications of DSD, and identify research priorities for further innovation in ART delivery.

## METHODS

### Mathematical Models

We adapted two previously validated agent-based models—*EMOD-HIV* (hereafter referred to as *EMOD*) and HIV *Synthesis* (hereafter referred to as *Synthesis*)—to simulate HIV epidemic trajectories and HIV health outcomes for each treatment delivery modality in four geographic settings. Details of the *EMOD* and *Synthesis* models, including calibration approaches, assumptions governing HIV transmission, HIV progression, and ART treatment, as well as DSD simulation scenarios, are reported in Supplementary Table 1 and elsewhere [16].

Briefly, *EMOD* is an HIV agent-based network transmission model that simulates heterosexual and mother-to-child HIV transmission using an age-structured sexual network [17], individual HIV disease and treatment trajectories, and a detailed HIV care and prevention continuum [18]. The model has been prospectively validated by comparing predicted results with empirical data from a multi-country community-based intervention before trial unblinding [16]. In this study, we employed *EMOD* to project HIV epidemics and health outcomes for Zambia (“Zambia/*EMOD*”), South Africa (“South Africa/*EMOD*”), and Malawi (“Malawi/*EMOD*”). We ran 250 model trajectories based on parameter sets optimized to match calibration targets for each country.

*Synthesis* tracks a simulated population of adults in LMICs across Eastern, Central, and Southern Africa (“LMICs/*Synthesis*”) [19]. Two hundred and fifty “setting scenarios” were developed to broadly represent a range of settings (eg, a country, district, town, or other small area) [19]. Each setting scenario was generated by one run of the model by sampling a set of parameter values, which reflects both the diversity of settings and uncertainties around model assumptions. In our analysis, we regard each of these 250 setting scenarios as one model trajectory.

### Model Adaptation for Differentiated Service Delivery Cost-Effectiveness Analysis

#### Effectiveness of Differentiated Service Delivery and Standard-of-Care

Our study focused on three DSD modalities: the community adherence group (CAG), a group-based healthcare worker-managed modality; urban adherence group (UAG), a group-based client-managed modality; and community HIV epidemic control model (HomeART), an individual community-based modality (Table 1 and Supplementary Appendix 1.1).

**Table 1.** Descriptions and Comparisons of Antiretroviral Therapy (ART) Delivery Modalities Simulated by EMOD-HIV and HIV Synthesis Models [15, 20]

Models	Standard Of Care (SoC)	Community Adherence Groups (CAGs)	Urban Adherence Groups (UAGs)	Community HIV Epidemic Control Model (HomeART)
Target patients	PLHIV aged ≥15 years old and have been on ART			
Providers	Clinical staff	Peers	Healthcare workers and community healthcare workers	Community health workers
Number of clinical/facility visits per year	4	2	2	1
Number of DSD visits per year	0	12	4	6
Location of ART resupply	Health facility	Community	Health facility	Home
frequency of ART dispensation	3-monthly	Members collect treatment for other CAG members during bi-yearly clinical visits, in a rotating manner	3-monthly with prepacked drugs dispensed during UAG meetings	First 3 months: monthly Thereafter: quarterly
Description of DSD visits	—	Group of +/− people meeting monthly at a community place	Group of 20–30 people meeting at health facilities and receiving adherence counseling by healthcare workers	People receive home visits for health screening, ART refills, and adherence monitoring

Abbreviation: DSD, differentiated service delivery.

In both models, the effectiveness of DSD modalities was represented by variations in the percentage decrease in annual rate at which patients stop ART in comparison to the SoC. We defined ART discontinuation as either LTFU (last visit missed >28 days) or stopping ART (last visit not missed by >28 days, but patient has stopped medication) [20, 21]. In our models, both outcomes were implemented as “stopping” ART, which was computed as 1 minus the annual retention rates for each modality, derived from a prior study that utilized Zambia's electronic health record data system (SmartCare) [20]. The baseline probabilities of stopping ART and of re-engaging in care after interruption varied by country and model (Supplementary Table 1). With the EMOD model, the annual interruption rates were 16.3% for South Africa and Malawi, and 3.6% for Zambia; with Synthesis, the annual interruption rates varied from 0.8% to 4.8% in the first year of ART, depending on setting scenarios and factors including drug toxicity, pregnancy, and duration on treatment [19]. The effectiveness of DSD in comparison to SoC was equal to the difference in the annual rate of stopping ART under SoC and the rate of stopping ART under DSD, divided by the annual rate of stopping ART under SoC. As a result, CAG reduced the annual ART stopping rate by 29% compared with SoC, UAG decreased it by 60%, and HomeART reduced it by 38% (Table 2 and Supplementary Table 2). Since the current DSD effectiveness was based on the Zambian setting, which may vary for different local contexts, we applied a broader range of estimates in the sensitivity analysis to account for uncertainties regarding the generalizability of the original study to external and future settings.

**Table 2.** Values and Ranges for Key Input Parameters for EMOD-HIV and HIV Synthesis Models

Parameter	Point Estimates		Ranges For Sensitivity Analysis	Reference
Effectiveness (reduction in 12-month ART stopping rate under DSD vs. SoC) ^a^				
SoC	Baseline		—	[20]
CAGs	29%		10%–75%	[20], assumed
UAGs	60%		10%–75%	[20]
Home ART delivery	38%		10%–75%	[20]
Cost (avg. cost per person-month, 2021 USD)^b^				
Zambia				
SoC	$13.88		—	[15]
CAGs	$16.31		$16.13–$18.01	[15]
UAGs	$20.49		$20.39–$22.20	[15]
HomeART	$20.51		$19.03–$25.80	[15]
South Africa				
SoC	$11.23		—	[15], adjusted
CAGs	$14.19		$13.79–$15.42	[15], adjusted
UAGs	$17.39		$17.28–$18.69	[15], adjusted
HomeART	$21.37		$19.06–$25.28	[15], adjusted
Malawi				
SoC	$10.67		—	[15], adjusted
CAGs	$12.52		$12.31–$13.83	[15], adjusted
UAGs	$15.74		$15.68–$17.11	[15], adjusted
HomeART	$15.68		$14.57–$19.82	[15], adjusted
Low- and middle-income countries (LMICs) in Africa				
SoC	$10.34		—	[15], adjusted
CAGs	$12.06		$11.87–13.34	[15], adjusted
UAGs	$15.19		$15.14–16.53	[15], adjusted
HomeART	$14.88		$13.87–18.91	[15], adjusted
Disability weights for living with HIV				
	*EMOD*	*Synthesis* ^c^		
HIV negative	0	0	—	[23]
HIV/AIDS with ART	0.078	0.03	0–0.111	[23]
HIV without ART	0.274	0.1	0.1–0.377	[23]

Abbreviations: CAGs, community adherence groups; HomeART, community HIV epidemic control model; SoC, standard of care; UAGs, urban adherence groups.

^a^DSD effectiveness compared to SoC = (the annual ART stopping rate in SoC—the annual ART stopping rate in DSD)/the annual ART stopping rate in SoC.

^b^Costs were inflated to 2021 USD values using country-specific inflation rates. Costs for South Africa, Malawi, and LMICs were adjusted based on the costs from the Zambia setting (Supplementary Appendix S1.2). The cost ranges for the sensitivity analysis were estimated under two extreme scenarios (ie, high-efficiency and low-efficiency scenarios). Further details are discussed in the sensitivity analysis section.

^c^In the *HIV Synthesis* model, DW for HIV/AIDS with ART and HIV without ART are further disaggregated into health states based on whether patients were experiencing drug toxicity, in WHO stage 3 or 4 conditions, and/or having TB co-infection (Supplementary Table S4 for detailed health states and disability weights).

#### Costs of Differentiated Service Delivery and Standard-of-Care

Costs of DSD and SoC were obtained from a previous ingredient-based costing analysis using Zambian SmartCare data over 2015–2017 [15], and are reported in Table 2. The cost analysis encompassed: facility-based clinical visits, pharmacy pickups, DSD visits, laboratory testing, and medications. We translated costs to South Africa, Malawi, and LMICs by adjusting the personnel costs for differences in local salaries. Details on cost assumptions are provided in Supplementary Appendix 1.2, with itemized cost breakdowns in Supplementary Table 3.

### Economic Evaluations

#### Disability-Adjusted Life-Years

We used disability-adjusted life-years (DALYs) to quantify the health burden of HIV, defined as years of life lost (YLLs) due to premature death plus years of healthy life lost due to living with a disability (YLDs) associated with HIV [22]. Disability weights were obtained from the Global Burden of Disease Study (GBD) 2019 (Table 2) [23]. Details on DALY calculations are provided in Supplementary Appendix 1.3.

#### Cost-Effectiveness Analysis

Our analysis adopted a health system perspective over a 40-year time horizon starting on January 01, 2022, focusing on PLHIV aged 15 and above who were receiving ART. We computed the incremental cost-effectiveness ratios (ICERs) of each DSD modality relative to SoC in each country/model setting and developed an optimal expansion frontier (ie, an efficient frontier) for DSD implementation. In the main analysis, we applied an annual discount rate of 3% per year to both costs and health impacts.

To contextualize ICERs, we compared them to benchmark CE thresholds, which represent the maximum ICER that would be considered cost-effective. The CE thresholds were derived in prior studies using different methodologies depending on whether HIV services were primarily domestically funded (in this study, South Africa) or primarily internationally funded through donor agencies (in this analysis, all other countries) [24]. For South Africa, the CE thresholds ranged from US$590 to US$3,525 per DALYs averted, based on the marginal opportunity cost of the national HIV program and the overall domestic healthcare expenditure, respectively. For Zambia, Malawi, and LMICs, the CE thresholds were set between US$500 and US$750, representing the marginal CE of donor-financed HIV services Africa, which exceeds the amount that could be afforded by domestic healthcare expenditures alone.

This study followed the Consolidated Health Economic Evaluation Reporting Standards 2022 (CHEERS 2022) Statement Checklist (Supplementary Appendix 2) [25]. All analyses were conducted in R version 4.3.1.

### Sensitivity Analysis

We conducted sensitivity analyses to account for uncertainties in key model assumptions, including DSD effectiveness, DSD costs, disability weights, discount rates, and the approach for calculating YLLs. We varied assumed DSD effectiveness at reducing ART discontinuation over the range 10%–75%, reflecting uncertainties in effectiveness when DSD is implemented in different programs and contexts [8]. For example, a cluster-randomized evaluation study in South Africa observed an absolute increase of 7.8% (95% CI: 2.1–13.6%) in 12-month retention after one year of adherence clubs vs. SoC [26]. The upper bound of this uncertainty interval is equivalent to a DSD effectiveness (relative increase in retention) of 75% in our models.

Disability weights were varied according to the uncertainty ranges reported in the GBD 2019 [23], with additional increases in uncertainty added to the lower-bound disability weight for health states of HIV with ART. Specifically, we tested reducing the lower bound of disability weight for HIV with ART to zero (from GBD's lower bound of 0.052), because GBD 2019 lists diarrhea as a medication side effect [23], which is an uncommon side effect of newer first-line medications used since 2018 (dolutegravir/tenofovir/lamivudine) [27]. We further examined the effect of harmonizing model assumptions regarding the disability weights of HIV infection. We obtained weighted average disability weights per year lived with HIV not on ART, and per year lived with HIV on ART, from the *Synthesis* model (Table 2 and Supplementary Table 4) and applied the same values in the *EMOD* model.

We conducted a bounding analysis of DSD costs, accounting for uncertainties related to resource allocation efficiency in response to missed appointments (Table 2 and Supplementary Table 5 for cost breakdowns). We consolidated uncertainties into two scenarios: a low-efficiency and a high-efficiency scenario. The low-efficiency scenario assumed that the health system incurred the full costs of DSD participation irrespective of patient engagement. This implied that costs of staff time, consumables (including medications), and infrastructure were not recuperated when patients missed scheduled visits or failed to receive prescribed medications. Conversely, the high-efficiency scenario assumed that the health system only bore costs corresponding to the actual resources utilized by patients, and all resources were efficiently re-directed to other services when patients did not attend scheduled visits or collect medications. In addition, we tested reducing the antiretroviral drug costs by 30% to represent potential reductions in drug costs.

Finally, we varied annual discount rates for costs and health benefits between 0% and 6%, varied our time horizon of analysis from 5 to 40 years, and tested an alternative approach to calculate DALYs averted over the time horizon (detailed in Supplementary Appendix 1.4).

## RESULTS

### Projected HIV Epidemics

**Figure 1.**
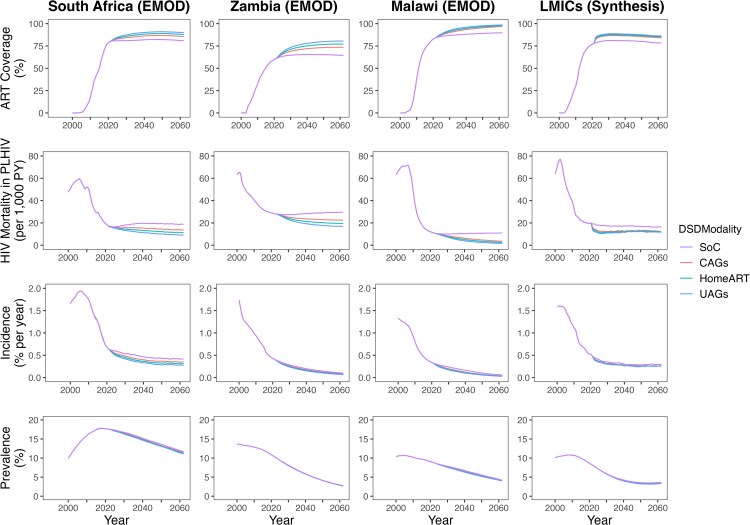
**ART coverage, HIV-related mortality, incidence, and prevalence from 2000 to 2062, by ART delivery modalities, geographic settings, and mathematical models.** Model simulations were conducted from 2022 to 2062; *x*-axis tick marks are shown at 10-year intervals for readability. Abbreviations: CAG, community adherence group; DSD, differentiated service delivery; HomeART, Community HIV epidemic control model; SoC, standard of care; UAG, urban adherence group.

Across all country/model settings, implementing DSD substantially increased ART coverage and reduced HIV-related mortality among PLHIV, and to a lesser extent, it reduced HIV incidence and prevalence (Figure 1). These effects were observed on top of diverse epidemic trajectories across *EMOD*-simulated countries (South Africa, Zambia, and Malawi), reflecting variations in baseline HIV epidemics across countries. Additionally, outputs from *Synthesis* modeling LMICs were in line with individual country outputs despite the diversity of model structures and assumptions included in the analysis.

### Cost-effectiveness of Differentiated Service Delivery

**Figure 2.**
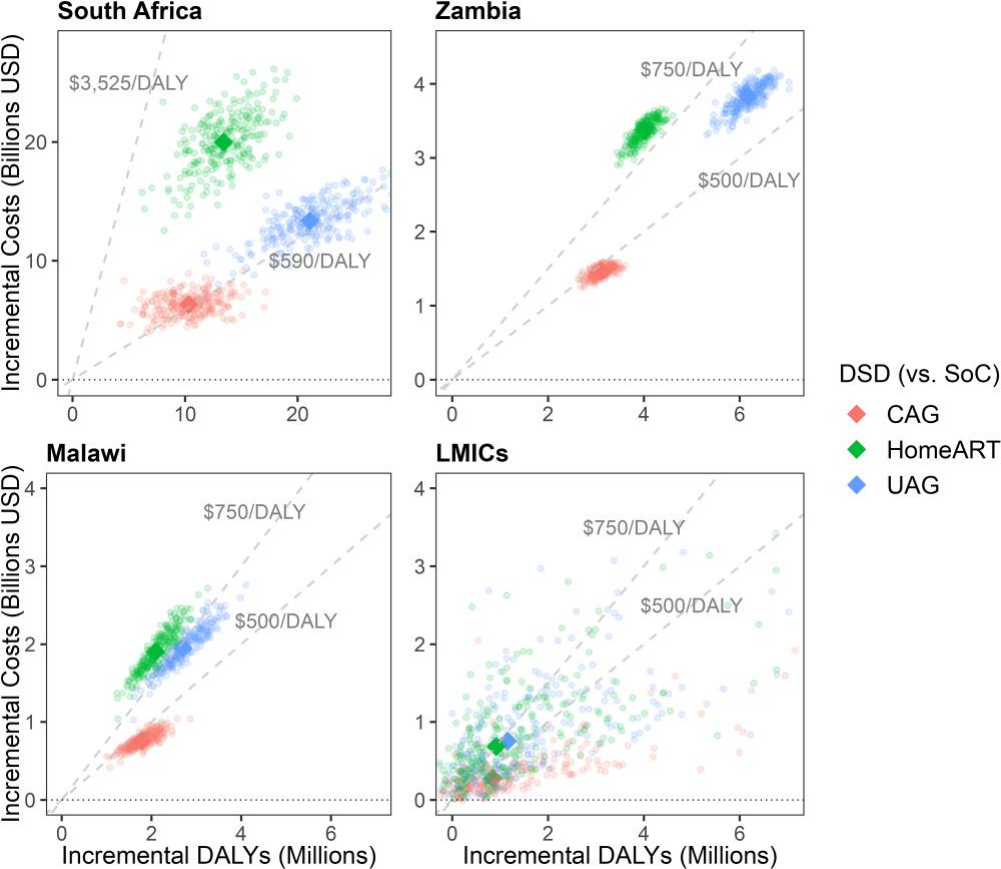
**Cost-effectiveness plane of incremental costs and incremental DALYs averted for DSDs compared with SoC over a 40-year simulation time horizon**, with cost-effectiveness threshold benchmarks shown for reference. Diamonds represented averaged model estimates, while small round dots represented individual model runs (n = 250). Abbreviations: CAG, community adherence group; DSD, differentiated service delivery; HomeART, Community HIV epidemic control model; SoC, standard of care; UAG, urban adherence group.

Over a 40-year time horizon, the most cost-effective DSD modality in all country/model settings was CAG (Figure 2 and Supplementary Table 6), with ICERs ranging between $274 and $604 per DALY averted, depending on the country/model. In all settings, UAG could achieve further impact beyond what is attainable by CAG, but at a higher cost per DALY averted: ranging between $590 and $720 per DALY averted, depending on the country/model. Consequently, the efficient frontier for DSD would begin with the implementation of CAG, followed by expansion to UAG if additional resources were available. In all settings, HomeART was dominated by UAG, as it averted fewer DALYs at higher costs. Therefore, HomeART did not appear on the efficient frontier for any settings.

The CE of DSD in each setting was influenced by the selection of the benchmark CE thresholds (Figure 2). In South Africa, all DSD modalities would be considered cost-effective relative to the upper CE threshold benchmarked at $3,525/DALY averted, but none would be considered cost-effective relative to the lower threshold benchmarked at $590/DALY averted. In Zambia, CAG and UAG, but not HomeART, met the criteria for CE at the upper CE threshold of $750/DALY averted, while CAG was also cost-effective at the lower threshold benchmarked at $500/DALY averted. Similarly, in Malawi, CAG was cost-effective at the lower threshold of $500/DALY averted and UAG was cost-effective at the upper threshold (750/DALY averted). In the *Synthesis* LMICs model, while CAG fell below the lower threshold of $500/DALY averted and the average ICER of UAG and HomeART was below the upper threshold of $750/DALY averted, approximately one-third of the bootstrap samples of UAG and HomeART had ICERs that exceeded the value of $750, reflecting the uncertainties in the setting scenarios and assumptions assumed by the *Synthesis* model.

### Sensitivity Analyses

**Figure 3.**
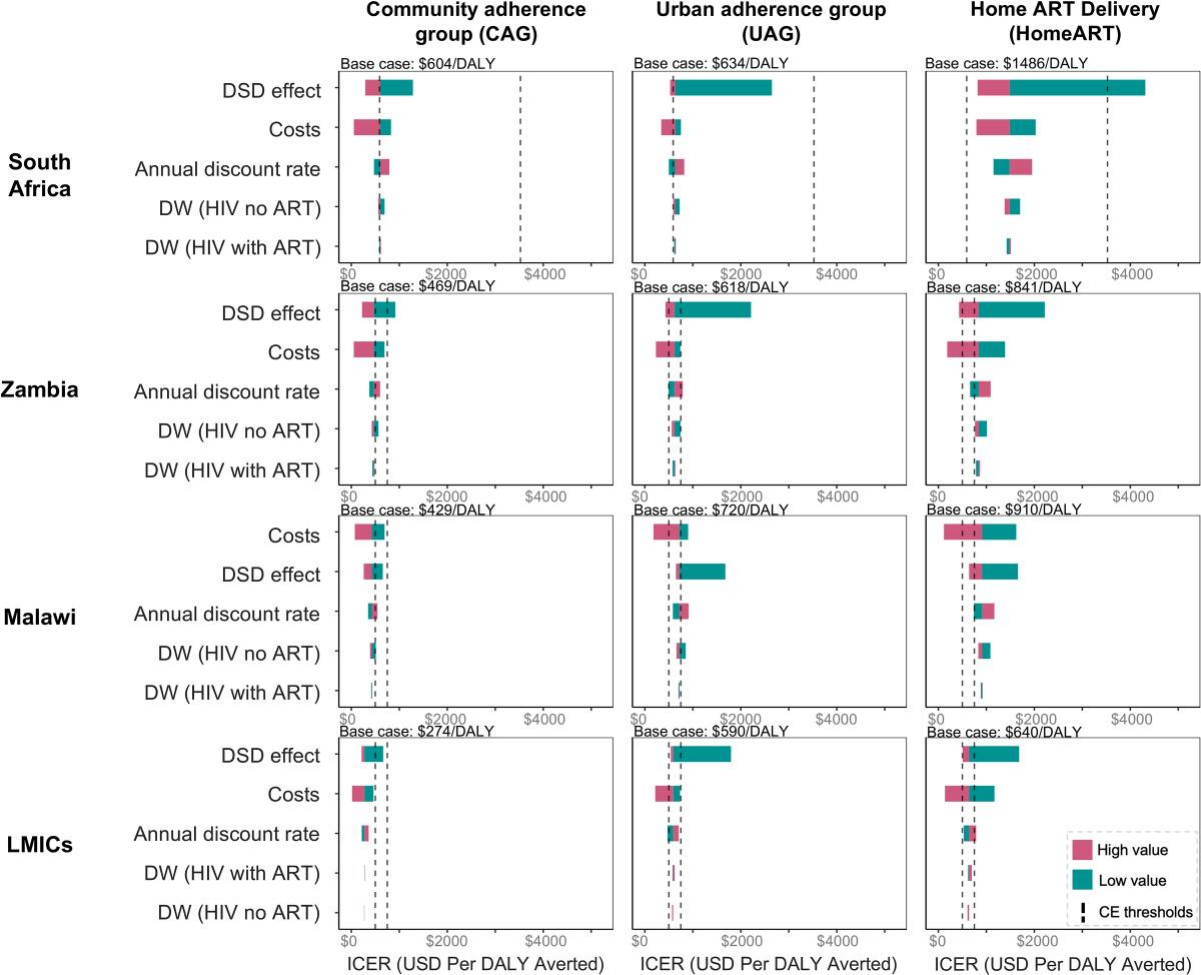
**One-way sensitivity analysis of DSD effectiveness, cost, disability weights, and annual discount rates.** The dashed vertical lines represented cost-effectiveness thresholds ($590 and $3525/DALY averted for South Africa, $500 and $750/DALY averted for Zambia, Malawi, and LMICs). Abbreviations: CAG, community adherence group; Costs, including two alternative extreme costing scenarios: low-efficiency scenario and high-efficiency scenario; DSD, differentiated service delivery; DSD effect, the effectiveness of DSD (ie, % reduction in ART discontinuation rate with DSD compared to the SoC); DW (HIV no ART), disability weights for PLHIV without ART; DW (HIV with ART), disability weights for PLHIV on ART; HomeART, Community HIV epidemic control model; UAG, urban adherence group.

The CE conclusions were most sensitive to DSD effectiveness in most settings (Figure 3). All DSD modalities were cost-effective at the upper CE threshold when effectiveness was 75% (ie, reducing the annual ART stopping rate by 75% compared with the SoC). Conversely, when DSD effectiveness was 10%, only CAG in LMICs, South Africa, and Malawi and UAG in South Africa were cost-effective with the upper CE thresholds.

In a bounding analysis of DSD costs, CE of all DSD improved substantially in the high-efficiency scenario, where resources associated with missed visits were efficiently allocated toward other services and the drug costs were reduced by 30%. Under these assumptions and compared with their lower CE thresholds, all modalities of DSD were cost-effective in all settings, except for HomeART in South Africa. Conversely, ICERs of all DSDs increased in the low-efficiency scenario, causing both UAG in Malawi and HomeART in LMICs to no longer be cost-effective when compared with the upper CE thresholds.

Assumptions related to disability weights had a minimal effect on the CE of DSD. The only DSD model that changed in CE was UAG in Malawi, which became not cost-effective with the lower-bound disability weight of HIV without ART. The estimated ICER of UAG in Malawi was close to the CE threshold in the main analysis, which allowed the uncertainty from disability weights to move this estimate across its CE threshold.

ICERs increased with higher annual discount rates. Under the upper CE thresholds, the CE of CAG remained robust to variation in discount rate between 0% and 6%, while UAG ceased to be cost-effective in Zambia and Malawi when the discount rate increased from 3% to 6%. Conversely, HomeART in Zambia and Malawi became cost-effective when the discount rate decreased from 3% to 0%.

## DISCUSSION

Using used empirical cost and effectiveness data to evaluate the CE of three DSD modalities using two well-established HIV epidemiological models. We found that DSD improved ART coverage, reduced HIV-related mortality and incidence, and was cost-effective for many combinations of models and settings (South Africa, Zambia, Malawi, and LMICs). Community adherence group was found to be most cost-effective DSD option in all country/model settings. When using higher CE thresholds benchmarked based on donor HIV funding (and general healthcare funding in South Africa), UAG was also cost-effective in all settings to further expand DSD. However, when using lower CE thresholds benchmarked based on domestic-only healthcare funding (and domestic HIV-specific financing in South Africa), CAG was not found to be cost-effective in South Africa (but was in other modeled settings), and UAG was only cost-effective in LMICs.

Our study adds to the accruing evidence on whether DSD and which DSD modality should be prioritized in Africa, even in cases where DSD makes HIV treatment provision more costly than SoC clinic-based care. Our findings differ from previous studies, likely due to updated treatment guidelines since previous studies were conducted (recommending universal ART eligibility), as well as updated data and simulation methods. Bango et al [28] utilized data from a year-long study in 2011, prior to guidelines for universal ART eligibility, and found that ART adherence club was cost-saving in South Africa from the health system perspective. This result would be less likely under the current analysis, which leveraged more recent findings that three DSD modalities are more costly than SoC. Sahu et al [29] found that a combined intervention of home-based testing and community ART was cost-effective from the health system perspective with an ICER of $102/DALY averted in KwaZulu-Natal, South Africa, using a mathematical model that forecast the HIV epidemic over 2020–2060. Their notably lower ICER may be attributable to the extremely high HIV prevalence in KwaZulu-Natal (27%), the relatively slower progress toward HIV treatment goals compared with other settings, and the added impact of HIV testing as part of the intervention that was analyzed.

Our study adds important insights regarding the CE of DSD implementation. Our finding that no DSD modalities were cost-effective over 5-year simulation horizon (Supplementary Figure 1) underscores the significance of ensuring sustainability in DSD implementation. Most DSD implementation research was restricted to a 5-year timeframe [8, 30], suggesting a need for continued studies to measure sustainability. Additionally, our finding that the CE of DSD was highly sensitive to the repurposing of resources from missed visits suggests the importance of a health system design that efficiently reallocates staff time, medications, and other resources when not utilized. Recent innovations in task-shifting and integration of HIV care with other in-demand health services, such as other sexual and reproductive health care and care for non-communicable diseases, may provide options for efficient implementation of both SoC and DSD [31].

Our study has several important limitations. First, in some settings, care guidelines restrict DSD to stable ART patients on ART for >6 months without complications [32]. We modeled DSD broadly to include all ART patients, including those who have initiated ART <6 months ago and those who have interrupted and resumed ART in the preceding 6 months. The importance of DSD for all ART patients has been recognized [12], and existing evidence has demonstrated the benefits of DSD to promote ART retention among those newly initiating ART [33], supporting the inclusive approach made in our forecasts. Second, our study did not incorporate the societal perspective due to the unavailability of high-quality patient cost data [14, 25, 30]. DSD has been shown to improve patient access to care by reducing travel expenses and the time spent on travel and waiting for appointments, which reduces lost wages and caregiving expenses [34]. These factors would likely reduce the total costs of DSD when analyzed from a societal perspective relative to a health system perspective. Third, patient preferences for DSD may vary based on individual needs. We acknowledge that not all population segments may be eligible or able to access all DSD modalities, e.g., UAG only being available for patients in more densely populated areas. Although our study suggests CAGs are an efficient initial expansion step, future research could explore scenarios that customize DSD expansion according to the needs and preferences of different population segments. Fourth, we made several simplifying assumptions to incorporate DSD effects and to parameterize input parameters. We captured the benefit of DSD as improvements in annual retention rate, yet we did not consider potential benefits on patient re-engagement, an area where data are sparser. This may underestimate the overall health benefits of DSD. Additionally, the local electronic health system where DSD costs were evaluated did not systematically track individual DSD visits [15]. As a result, estimated costs were not directly extracted from medical records but were calculated by the authors. We conducted a bounding analysis on cost across the plausible range given these uncertainties. Finally, we recognize our sensitivity analyses may not incorporate all uncertainties inherent in a modeling study. Nevertheless, we considered uncertainties across multiple dimensions, including baseline HIV epidemics, mathematical model assumptions, cost assumptions, treatment effects, and economic thresholds. A strength of our study is the use of two mathematical models in four settings to test the robustness of findings against variations in models and settings. Our study could be further refined after the conclusion of an ongoing observational study of DSD in South Africa, Malawi, and Zambia, which collects more detailed provider and patient costs associated with DSD modalities through time-and-motion observations, chart review, and interviews [35].

DSD has the potential to substantially increase the health benefits of ART in Africa. Our analysis supports the expansion of CAG and UAG as cost-effective alternatives to SoC for HIV treatment delivery, depending on relevant CE thresholds. ICERs derived from this study can be used for comparisons with alternative HIV services, assisting decision-making regarding the allocation of limited resources to maximize health benefits and accelerate progress in alleviating the burden of HIV/AIDS in resource-constrained settings.

## Supplementary Material

ofag257_Supplementary_Data

## Supplementary Data

Supplementary materials are available at *Open Forum Infectious Diseases* online. Consisting of data provided by the authors to benefit the reader, the posted materials are not copyedited and are the sole responsibility of the authors, so questions or comments should be addressed to the corresponding author.
