# Supplementary material for: Cost-Effectiveness of Differentiated Service Delivery for HIV Treatment: A Combined Mathematical Modeling Study of Four African Settings
Source: Open Forum Infect Dis. 2026 Jun 19;13(6):ofag257. doi: 10.1093/ofid/ofag257 (PMC13280640; doi:10.1093/ofid/ofag257)
Supplement: ofag257_Supplementary_Data [file ofag257_supplementary_data.docx]

**Supplementary Materials**

**Cost-effectiveness of differentiated service delivery for HIV treatment: A combined mathematical modeling study of four African settings**

Shiying You, MPH,^a^ Hae-Young Kim, PhD, ^a^ Andrew N. Phillips, PhD,^b^ Daniel T. Citron, PhD,^a^ David Kaftan, MS,^a^ Ingrida Platais, MSc,^a^ Loveleen Bansi-Matharu, PhD,^b^ Valentina Cambiano, PhD,^b^ Brooke E. Nichols, PhD,^c^ Youngji Jo, PhD,^d^ Ronald S. Braithwaite, PhD,^a^ Edinah Mudimu, PhD,^e^ Anna Bershteyn, PhD^a*^

^a^ Department of Population Health, NYU Grossman School of Medicine, Translational Research Building, Floor 6, New York, NY 10016, USA.

^b^ Institute for Global Health, University College London, 90 Tottenham Court Road, London W1T 4TJ, UK.

^c^ FIND, Chemin du Pommier 40, 1218 Grand-Saconnex, Switzerland.

^d^ Department of Public Health Sciences, School of Medicine, University of Connecticut, 200 Academic Wy, Farmington, CT 06032, USA.

^e^ Department of Decision Sciences, University of South Africa, Preller St, Muckleneuk, Pretoria, 0002, South Africa.

*Correspondence to: Anna Bershteyn ([Anna.Bershteyn@nyulangone.org](mailto:Anna.Bershteyn@nyulangone.org)), Department of Population Health, NYU Grossman School of Medicine, 180 Madison Ave, Floor 17 New York, NY 10016, USA

Alternative correspondence: Shiying You ([Shiying.You@nyulangone.org](mailto:Shiying.You@nyulangone.org)), Department of Population Health, NYU Grossman School of Medicine, 180 Madison Ave, Floor 17 New York, NY 10016, USA

## **S1. Supplementary methods**

**Overview**

This study integrated data on the effectiveness and costs of four ART delivery modalities and simulated epidemiological models of four geographic settings using two previously validated HIV individual-based transmission models. Outputs for each delivery modality in each modeled geographic setting were used to calculate HIV incidence, prevalence, HIV-related mortality, ART coverage, disability-adjusted life years (DALYs), and costs from 2022 to 2062. In addition, in each geographic setting, we calculated the incremental cost-effectiveness ratios (ICERs) of each DSD modality compared to SoC and developed an optimal expansion frontier (i.e., an efficient frontier) for DSD implementation.

### **S1·1 Detailed description of DSD modalities**

Our study focused on three DSD modalities: the community adherence group (CAG), a group-based healthcare worker-managed modality, urban adherence group (UAG), a group-based client-managed modality, and community HIV epidemic control model (HomeART), an individual community-based modality. These modalities were modeled in four geographic settings: South Africa, Malawi, Zambia, and a collective representation of African low- and middle-income countries (LMICs). DSD modalities were distinguished by multiple factors: the cadre of providers delivering ART (clinical staff for SoC, community health workers or lay volunteers for DSD), the number of annual clinic visits (4 for SOC, 1-2 for DSD), the number of annual DSD visits (4-12, varying by modality), the location of ART resupply (health facility, community, or home), and the frequency of ART dispensation (3-monthly for SOC and 1-6 monthly for DSD), as detailed in Table 1.

### **S1·2 Details of ingredient-based DSD costing**

Patient attendance at DSD sessions was inferred by multiplying the recommended number of sessions for each DSD modality by the percentage of clinic visits attended, as DSD attendance was not directly recorded in SmartCare.^S1^ Further uncertainty existed regarding whether the cost of a missed visit was recuperated by the health system. Specifically, there was uncertainty about whether personnel time and resources were redirected to alternative activities or whether the full cost of an appointment was incurred regardless of attendance. In our main analysis, we assumed that the resources (personnel time, consumables, infrastructure, and other operational needs) associated with a missed visit would be expended. However, we assumed that the cost of medications would be saved if they were not dispensed to the patient. In the sensitivity analysis, we further explored two alternative costing scenarios to reflect the potential cost variations associated with missed DSD visits. However, costs of SoC remained consistent across both scenarios, as this modality does not incorporate DSD visits.

We subdivided costs of ART delivery modalities into three subcategories: unit cost per visit, laboratory testing, and medications (Supplementary Figure S2). The unit cost per visit covered all resources utilized to provide ART-delivery-related services, primarily including personnel resources (health worker salaries, etc.), equipment, and other operational needs (travel, etc.).

We adjusted the annual average cost per patient for South Africa, Malawi, and LMICs by modifying the personnel costs, based on the salary ratios of healthcare personnel at comparable levels in Zambia and the respective countries.^S2-S4^ Country-specific healthcare worker salaries were estimated by multiplying the gross domestic product (GDP) and the world bank healthcare worker earning indices (Supplementary Table S7).^S2-S4^ For the aggregate LMICs setting simulated by *HIV* *Synthesis*, we used GDP for “Sub-Saharan Africa, excluding high-income”.^S2-S4^ The salary ratios were then calculated for South Africa, Malawi, and LMICs in comparison to Zambia to facilitate this adjustment. Specifically:

$${salary}_{i, t}=Average Earning Index_{t}\times GDP_{i,t}$$

$$ratio_{i,Zambia}=\frac{wage_{i,t}}{wage_{Zambia,t}}$$

$i$: country of interest.

$t$: year. In this study, we used 2010 because the average earning index was estimated in 2010 USD.^S2^

$Average Earning index_{t}$: the multiplier of GDP per capita used to estimate country-specific health worker earning in year $t$.

$GDP_{i,t}$: the GDP per capita for country $i$ in year $t$.

Other cost components, including equipment, lab testing, and medications were assumed to be consistent across our simulated settings. Our simulated settings generally use the same generic supplier for equipment and medications. In addition, Nichols et al. compared costs of lab testing between Zambia and South Africa, which found the public sector prices were approximately the same, supporting our assumption of consistent lab testing costs across our simulated setting.^S1^

All costs were inflated to 2021 USD values based on country-specific inflation rates.

### **S1·3 Details of DALY calculations**

We used DALYs to quantify the health burden of HIV, defined as years of life lost (YLLs) due to premature death plus years of healthy life lost due to living with a disability (YLDs) associated with HIV^S5^ in adults (15+) with HIV who were receiving ART. YLLs included each person-year that an individual was not alive due to HIV but would have been alive had they not had HIV. YLLs were accrued in each year until the end of the life expectancy without HIV, which was assumed to be 80 years, or the end of the time horizon of analysis, whichever occurred earlier. YLDs were calculated as the product of disability weights for various health states and the durations that patients spent in these health states. For *EMOD-HIV*, health states causing decrements in quality of life included having an HIV infection, with or without treatment. For *HIV* *Synthesis*, the considered health states involved experiencing drug toxicity from treatment, being in HIV WHO stage 3 or 4 conditions, and having TB co-infections (Supplementary Table S4).^S6^

### **S1·4 Sensitivity analysis: alternative approach to calculating DALYs over the time horizon**

We tested the effect of incorporating all years of life lost (YLLs) between the year of HIV-related death and the expected life expectancy without HIV, even if some years of life lost occur after the end of the simulation duration. This method deviates from typical health economic approaches that restrict health impacts to the time horizon of analysis, but it has been used in some recent HIV economic analyses to more fully capture long-term health effects.^S7^ We tested this alternative approach in settings simulated in *EMOD-HIV*: Zambia, Malawi, and South Africa.

Under this alternative method for YLL calculation, all DSD modalities were cost-effective at the upper CE threshold benchmark for South Africa, Zambia, and Malawi (Supplementary Table S8).

## **S2. The Consolidated Health Economic Evaluation Reporting Standards 2022 (CHEERS 2022) Statement Checklist**

| **Topic** | **No.** | **Item** | **Location where item is reported** |
| --- | --- | --- | --- |
| **Title** |  |  |  |
|  | 1 | Identify the study as an economic evaluation and specify the interventions being compared. | The title indicates that the intervention is 'differentiated service delivery for HIV treatment,' and the term 'cost-effectiveness' indicates that it is an economic evaluation study. |
| **Abstract** |  |  |  |
|  | 2 | Provide a structured summary that highlights context, key methods, results, and alternative analyses. | The abstract includes four major sections: background (for context), methods (for key methodologies and sensitivity analyses, as part of the alternative analyses), findings (key results), and interpretation. |
| **Introduction** |  |  |  |
| **Background and objectives** | 3 | Give the context for the study, the study question, and its practical relevance for decision making in policy or practice. | The first paragraph provides context on the current HIV burden and the key challenges of HIV treatment. The second paragraph introduces the rationale and key principles of differentiated service delivery (DSD). The third paragraph highlights the major gaps in DSD within study settings, where additional research is needed. The last paragraph discusses the practical relevance for decision-making regarding the expansion of DSD in Africa. |
| **Methods** |  |  |  |
| **Health economic analysis plan** | 4 | Indicate whether a health economic analysis plan was developed and where available. | The economic evaluation plan was discussed in detail in the “economic evaluations” section and Supplementary Appendix S1. |
| **Study population** | 5 | Describe characteristics of the study population (such as age range, demographics, socioeconomic, or clinical characteristics). | Described in “Economic Evaluations” section. |
| **Setting and location** | 6 | Provide relevant contextual information that may influence findings. | Our study considered four African geographic settings using two mathematical models, acknowledging the potential significant impact of context on our results. Specifically, the CE of DSD is likely to vary based on different baseline HIV epidemics, as detailed in the "mathematical models" subsection. Additionally, variations in DSD costs, influenced by factors such as local productivity levels and other economic conditions, were explored in the "costs of DSD and SoC" subsection. |
| **Comparators** | 7 | Describe the interventions or strategies being compared and why chosen. | Discussed in “model adaptation for DSD CEA” section. |
| **Perspective** | 8 | State the perspective(s) adopted by the study and why chosen. | Health system perspective (in the “economic evaluations” section)  We acknowledged the lack of societal perspective as a limitation in “discussion”. |
| **Time horizon** | 9 | State the time horizon for the study and why appropriate. | Discussed in the “economic evaluations” section. |
| **Discount rate** | 10 | Report the discount rate(s) and reason chosen. | Discussed in the “economic evaluations” section. |
| **Selection of outcomes** | 11 | Describe what outcomes were used as the measure(s) of benefit(s) and harm(s). | Discussed in the “economic evaluations” section. |
| **Measurement of outcomes** | 12 | Describe how outcomes used to capture benefit(s) and harm(s) were measured. | Discussed in the “economic evaluations” section. |
| **Valuation of outcomes** | 13 | Describe the population and methods used to measure and value outcomes. | The valuation of outcomes was made by making comparisons between the intervention and the reference strategy (standard of care), which was discussed in the “Model adaptation for DSD CEA” section. |
| **Measurement and valuation of resources and costs** | 14 | Describe how costs were valued. | The incremental costs of interventions were transformed as incremental cost-effectiveness ratios, which were compared to cost-effectiveness thresholds, as discussed in the 'economic evaluations' section |
| **Currency, price date, and conversion** | 15 | Report the dates of the estimated resource quantities and unit costs, plus the currency and year of conversion. | Discussed in the “costs of DSD and SoC” section and Supplementary Appendix S1. |
| **Rationale and description of model** | 16 | If modelling is used, describe in detail and why used. Report if the model is publicly available and where it can be accessed. | Two previously validated network-based HIV models were used to project the epidemiological outcomes under intervention and baseline scenarios, which was discussed in the “mathematical models” section. |
| **Analytics and assumptions** | 17 | Describe any methods for analysing or statistically transforming data, any extrapolation methods, and approaches for validating any model used. | Assumptions for mathematical models were detailed in “Mathematical models” section and Supplementary Appendix S1. We also adjusted costs based on local productivity and healthcare worker salaries, detailed in the 'costs of DSD and SoC' section and Supplement Appendix S1. |
| **Characterising heterogeneity** | 18 | Describe any methods used for estimating how the results of the study vary for subgroups. | We adapted two mathematical models, to account for different modeling assumptions governing HIV transmission, HIV progression, and ART treatment by patient characteristics and HIV status. We also evaluated the intervention's impacts across various geographic settings. Specifically, our analysis accounted for variations in local HIV burden and transmission rates, factoring in diverse patient characteristics such as sex and risky sexual behaviors within these regions. |
| **Characterising distributional effects** | 19 | Describe how impacts are distributed across different individuals or adjustments made to reflect priority populations. | In the “Mathematical Models” section, we described how we evaluated the impact of DSD across different geographic settings through two mathematical models. Recognizing that HIV affects a broad range of populations, our study focused on the general population to explore the cost-effectiveness of scaling up the treatment intervention at a national level for all PLHIV. |
| **Characterising uncertainty** | 20 | Describe methods to characterise any sources of uncertainty in the analysis. | Discussed in the “sensitivity analysis” section. |
| **Approach to engagement with patients and others affected by the study** | 21 | Describe any approaches to engage patients or service recipients, the general public, communities, or stakeholders (such as clinicians or payers) in the design of the study. | This study, a secondary data analysis using a mathematical modeling approach, indirectly engaged with patients, service recipients, and other key stakeholders through utilizing data from published studies. The original studies informing the effectiveness and costs of DSD had direct interaction with key stakeholders, mainly health care staff and the ministry of health. The models employed in our study were previously validated and developed with the support and input of these stakeholders. While our direct engagement in the study's design was limited due to the nature of secondary data analysis, the insights and validations provided by stakeholders in the initial studies were crucial in shaping the parameters and assumptions used in our analysis. |
| **Results** |  |  |  |
| **Study parameters** | 22 | Report all analytic inputs (such as values, ranges, references) including uncertainty or distributional assumptions. | Table 2. |
| **Summary of main results** | 23 | Report the mean values for the main categories of costs and outcomes of interest and summarise them in the most appropriate overall measure. | Figure 2 and “Results” section. |
| **Effect of uncertainty** | 24 | Describe how uncertainty about analytic judgments, inputs, or projections affect findings. Report the effect of choice of discount rate and time horizon, if applicable. | Figure 3 and “sensitivity analysis” sections under “Results”. |
| **Effect of engagement with patients and others affected by the study** | 25 | Report on any difference patient/service recipient, general public, community, or stakeholder involvement made to the approach or findings of the study | This study is a secondary data analysis utilizing data on DSD models from previously published papers. Both effectiveness and cost data were sourced from the national electronic health system with interactions with stakeholders, including the ministry of health. This enabled us to understand the current utilization and impacts of DSD for people living with HIV after its rollout in the country, supporting our objective to evaluate the cost-effectiveness of scaling up these models at a national level. |
| **Discussion** |  |  |  |
| **Study findings, limitations, generalisability, and current knowledge** | 26 | Report key findings, limitations, ethical or equity considerations not captured, and how these could affect patients, policy, or practice. | Key findings: first paragraph.  Limitations were discussed in the second last paragraph. Equity considerations were discussed as the first limitation point.  Potential public impacts: first and last paragraph. |
| **Other relevant information** |  |  |  |
| **Source of funding** | 27 | Describe how the study was funded and any role of the funder in the identification, design, conduct, and reporting of the analysis | Detailed in “role of funding source” and “acknowledge”. |
| **Conflicts of interest** | 28 | Report authors conflicts of interest according to journal or International Committee of Medical Journal Editors requirements. | Detailed in the “declaration of interests” section. |

## **Supplementary Tables**

**Table S1. Major characteristics of EMOD-HIV and HIV Synthesis models.^24^**

|  | ***EMOD-HIV*** | ***HIV Synthesis*** |
| --- | --- | --- |
| **Bootstrap samples** | 250 | 250 |
| **Target population** | Country-specific models: Zambia, South Africa, Malawi | A simulated population of adults in African LMICs, accounting for both inter- and intra-country uncertainties. |
| **Calibration approach** | Country-specific calibration was based on country-specific data on population size, age and sex-specific HIV incidence, HIV prevalence, and ART coverage. The data were from 2018 for Malawi, 2011 for South Africa, and 2015 for Zambia. | Varied across different setting scenarios in terms of population characteristics, sexual behavior, HIV-related factors, and ART; no calibration was performed to a specific country. |
| **Transmission structure** | Age and sex-structured network for coital acts and childbirths | A stochastic process driven by dyadic partnerships, integrating partnership stratification (long-term vs. short-term), viral load-dependent infectiousness, and biological and behavioral risk modifiers (e.g., age-gender mixing) * |
| **Untreated HIV disease progression** | Age-dependent rate of CD4 cell count decrease | Viral load gradually increases over time, with a small effect of sex on this trajectory; CD4 varied based on the latest viral load; AIDS rate based on latest CD4, viral load, age |
| **Effect of ART** | CD4 cell count recovery and viral suppression lead to mortality and HIV transmission reductions | CD4 cell count recovery and viral load suppression lead to mortality and HIV transmission reductions |
| **Baseline rate of ART interruption** | Varies by factors including sex and year, which corresponds to changes in HIV treatment policies. Across simulated countries, the annual probabilities are 16.3% for South Africa and Malawi, and 3.6% for Zambia | Varies across different setting scenarios and by pregnancy status and HIV treatment factors (ART adherence, ART toxicity, and time on ART), reflecting the effects of current policies that offer flexible alternative models to reduce the risk of ART interruption |
| **Baseline rate of ART re-initiation** | Varies by age, sex, pregnancy status, CD4 cell count, and AIDS symptoms; does not differ by prior treatment history. Across simulated countries, the annual probabilities are 90% for South Africa and Zambia, and 95% for Malawi | Varies across different setting scenarios and by factors such as pregnancy, sexual behaviors, and HIV symptoms, reflecting the effects of current policies to increase the probability of re-engaging with care and ART |
| **ART delivery modality scenarios and approach to incorporate the effective of DSD into model** | SoC (reference),  CAG, UAG, and Home ART  Incorporate the effect of DSD by reducing the baseline rate of ART interruption by 29% for CAG, 38% for HomeART, and 60% for UAG. | |
| **DALYs calculation** | The sum of YLLs and YLDs. YLLs was incurred each year since death, assuming a life expectancy of 80 years. The health states considered for YLDs include HIV with and without treatment | The sum of YLLs and YLDs. YLLs was incurred each year since death, assuming a life expectancy of 80 years. The health states considered for YLDs include experiencing drug toxicity from treatment, being in HIV WHO stage 3 or 4 conditions, and having TB co-infections |

Abbreviations: SoC: standard of care; CAGs: community adherence groups; UAGs: urban adherence groups; HomeART: Community HIV epidemic control model; YLLs: years of life lost due to premature death; YLDs: years of healthy life lost due to living with a disability.

* For further details on the HIV transmission structure of *HIV Synthesis*, refer to the HIV Synthesis model documentation (available at: hivmodeling.org/model-database/hiv-synthesis), specifically Sections 3.5–3.7.

**Table S2. Adaptation of patient retention data from the Zambian observational study into DSD effectiveness for *EMOD-HIV* and *HIV* *Synthesis* models.**

| **Modality name** | **47 months retention rate from *Jo et al*.** | **Annual ART stopping rate** | **DSD effectiveness implemented in *EMOD-HIV* and *HIV* *Synthesis* (% decrease in annual ART stopping rate compared to SoC)** |
| --- | --- | --- | --- |
| SoC | 86·57% | 3·62% | Reference |
| CAG | 90·31% | 2·57% | 29% |
| UAG | 94·48% | 1·44% | 60% |
| HomeART | 91·56% | 2·23% | 38% |
| Equation of DSD effectiveness compared to SoC = (annual ART stopping rate in SoC –annual ART stopping rate in DSD) / the annual ART stopping rate in SoC | | | |

**Table S3. Breakdowns of the average cost per person-year by countries for main analysis.** All costs were calculated in year 2018 and inflated to 2021 based on country-specific inflation rates. Data for Zambia retrieved from Nichol et al. (2021),^S1^ and data for other countries were adjusted by method described in Supplementary Appendix S1·2.

|  | **CAG** | **UAG** | **HomeART** | **SoC** |  | **CAG** | **UAG** | **HomeART** | **SoC** |
| --- | --- | --- | --- | --- | --- | --- | --- | --- | --- |
| **Zambia** | | | | | **South Africa** | | | | |
| Facility visit | 5·63 | 4·87 | 1·10 | 4·99 | Facility visit | 12·03 | 10·41 | 2·35 | 10·67 |
| Pharmacy pickup | 4·00 | 6·29 | 2·60 | 4·32 | Pharmacy pickup | 8·83 | 13·88 | 5·74 | 9·53 |
| DSD visits | 11·93 | 11·26 | 51·44 | 0·00 | DSD visits | 23·32 | 22·28 | 110·37 | 0·00 |
| Laboratory testing | 6·92 | 23·24 | 4·56 | 4·61 | Laboratory testing | 6·92 | 23·24 | 4·56 | 4·61 |
| Non-antiretroviral drugs | 0·10 | 0·18 | 0·18 | 0·13 | Non-antiretroviral drugs | 0·10 | 0·18 | 0·18 | 0·13 |
| Antiretroviral drugs | 89·01 | 101·87 | 87·96 | 86·04 | Antiretroviral drugs | 89·01 | 101·87 | 87·96 | 86·04 |
| Total (2018) | 117·59 | 147·71 | 147·84 | 100·09 | Total (2018) | 140·21 | 171·86 | 211·16 | 110·98 |
| Inflated to 2021 | 181·25 | 227·68 | 227·88 | 154·28 | Inflated to 2021 | 157·67 | 193·26 | 237·45 | 124·80 |
| **Malawi** | | | | | **LMICs** | | | | |
| Facility visit | 5·45 | 4·71 | 1·06 | 4·83 | Facility visit | 5·07 | 4·39 | 0·99 | 4·50 |
| Pharmacy pickup | 3·91 | 6·15 | 2·54 | 4·22 | Pharmacy pickup | 3·61 | 5·67 | 2·34 | 3·89 |
| DSD visits | 11·72 | 11·06 | 50·37 | 0·00 | DSD visits | 11·00 | 10·36 | 46·63 | 0·00 |
| Laboratory testing | 6·92 | 23·24 | 4·56 | 4·61 | Laboratory testing | 6·92 | 23·24 | 4·56 | 4·61 |
| Non-antiretroviral drugs | 0·10 | 0·18 | 0·18 | 0·13 | Non-antiretroviral drugs | 0·10 | 0·18 | 0·18 | 0·13 |
| Antiretroviral drugs | 89·01 | 101·87 | 87·96 | 86·04 | Antiretroviral drugs | 89·01 | 101·87 | 87·96 | 86·04 |
| Total (2018) | 117·11 | 147·21 | 146·67 | 99·83 | Total (2018) | 115·71 | 145·71 | 142·66 | 99·17 |
| Inflated to 2021 | 139·12 | 174·88 | 174·24 | 118·60 | Inflated to 2021 | 134·05 | 168·81 | 165·28 | 114·89 |

**Table S4. Disability weight assumptions for HIV-related health states in *EMOD-HIV* and *HIV Synthesis* models.**

| ***Comparisons of assumptions regarding the disability weights of HIV infection*** | | | | | |
| --- | --- | --- | --- | --- | --- |
| ***EMOD-HIV*** | | ***HIV Synthesis*** | | | |
| **Health states** | **Disability weights** | **Health states** | | | **Disability weights** |
| HIV negative | 0 | On ART with any drug toxicity | | | 0·05 (0·25 if greater toxicity) |
| HIV without ART | 0·274 | Any WHO stage 3 condition (except TB) | | | 0·22 |
| HIV/AIDS with ART | 0·078 | WHO stage 3 condition with TB | | | 0·40 |
|  | | Any WHO stage 4 condition | | | 0·54 |
|  |  | Otherwise | | | 0 |
| ***Harmonizing model assumptions regarding the disability weights of HIV infection by treatment status*** | | | | | |
| **Health states** | | | **Disability weights** | **Proportions** | |
| **HIV with ART** | | | | | |
| People on ART with no 1) drug toxicity or 2) current WHO stage 3 or 4 conditions | | | 0 | 83% | |
| People with a current drug toxicity | | | 0·15 | 17% | |
| People with WHO stage 3 event (apart from TB) in the period | | | 0·22 | Negligible because they only last about three months and are very rare among patients on ART. | |
| People with WHO stage 3 event with active TB | | | 0·40 |  |  |
| People with AIDS/WHO stage 4 event | | | 0·54 |  |  |
| Weighted average for “HIV with ART” | | | **0·03** | | |
| **HIV without ART** | | | | | |
| People with no ART (asymptomatic) | | | 0 | 70% | |
| People with no ART and low CD4 count + WHO stage 3 event (some is TB) (around 3 months) | | | 0·27 | 25% | |
| People with no ART and low CD4 count + WHO stage 4 event | | | 0·54 | 5% | |
| Weighted average for “HIV without ART” | | | **0·10** | | |

**Table S5. Breakdowns of the average cost per person-year by countries** **for the one-way sensitivity analysis.** All costs were calculated in year 2018 and inflated to 2021 based on country-specific inflation rates. Data for Zambia retrieved from Nichol et al. (2021),^S1^ and data for other countries were adjusted by method described in Supplementary Appendix S1·2.

| **Low-efficiency scenario** | **CAG** | **UAG** | **HomeART** | **SoC** | **High-efficiency scenario** | **CAG** | **UAG** | **HomeART** | **SoC** |
| --- | --- | --- | --- | --- | --- | --- | --- | --- | --- |
| **Zambia** | | | | | **Zambia** | | | | |
| Facility visit | 5·63 | 4·87 | 1·1 | 4·99 | Facility visit | 5·63 | 4·87 | 1·1 | 4·99 |
| Pharmacy pickup | 4 | 6·29 | 2·6 | 4·32 | Pharmacy pickup | 4 | 6·29 | 2·6 | 4·32 |
| DSD visits | 11·93 | 11·26 | 51·44 | 0 | DSD visits | 9·92 | 10·68 | 40·78 | 0 |
| Laboratory testing | 6·92 | 23·24 | 4·56 | 4·61 | Laboratory testing | 6·92 | 23·24 | 4·56 | 4·61 |
| Non-antiretroviral drugs | 0·1 | 0·18 | 0·18 | 0·13 | Non-antiretroviral drugs | 0·1 | 0·18 | 0·18 | 0·13 |
| Antiretroviral drugs | 101·21 | 114·66 | 126·63 | 86·04 | Antiretroviral drugs | 62·31 | 71·31 | 61·57 | 60·23 |
| Total (2018) | 129·79 | 160 | 186 | 100·09 | Total (2018) | 88·88 | 116·57 | 110·79 | 74·28 |
| Inflated to 2021 | 200·06 | 246·62 | 286·70 | 154·28 | Inflated to 2021 | 136·99 | 179·68 | 170·77 | 114·49 |
| **South Africa** | | | | | **South Africa** | | | | |
| Facility visit | 12·03 | 10·41 | 2·35 | 10·67 | Facility visit | 12·03 | 10·41 | 2·35 | 10·67 |
| Pharmacy pickup | 8·83 | 13·88 | 5·74 | 9·53 | Pharmacy pickup | 8·83 | 13·88 | 5·74 | 9·53 |
| DSD visits | 23·32 | 22·28 | 110·37 | 0 | DSD visits | 19·39 | 21·13 | 87·5 | 0 |
| Laboratory testing | 6·92 | 23·24 | 4·56 | 4·61 | Laboratory testing | 6·92 | 23·24 | 4·56 | 4·61 |
| Non-antiretroviral drugs | 0·1 | 0·18 | 0·18 | 0·13 | Non-antiretroviral drugs | 0·1 | 0·18 | 0·18 | 0·13 |
| Antiretroviral drugs | 101·21 | 114·66 | 126·63 | 86·04 | Antiretroviral drugs | 62·31 | 71·31 | 61·57 | 60·23 |
| Total (2018) | 152·41 | 184·65 | 249·83 | 110·98 | Total (2018) | 109·58 | 140·15 | 161·90 | 85·17 |
| inflated to 2021 | 171·39 | 207·64 | 280·94 | 124·80 | inflated to 2021 | 123·22 | 157·60 | 182·06 | 95·77 |
| **Malawi** | | | | | **Malawi** | | | | |
| Facility visit | 5·45 | 4·71 | 1·06 | 4·83 | Facility visit | 5·45 | 4·71 | 1·06 | 4·83 |
| Pharmacy pickup | 3·91 | 6·15 | 2·54 | 4·22 | Pharmacy pickup | 3·91 | 6·15 | 2·54 | 4·22 |
| DSD visits | 11·72 | 11·06 | 50·37 | 0 | DSD visits | 9·75 | 10·49 | 39·93 | 0 |
| Laboratory testing | 6·92 | 23·24 | 4·56 | 4·61 | Laboratory testing | 6·92 | 23·24 | 4·56 | 4·61 |
| Non-antiretroviral drugs | 0·1 | 0·18 | 0·18 | 0·13 | Non-antiretroviral drugs | 0·1 | 0·18 | 0·18 | 0·13 |
| Antiretroviral drugs | 101·21 | 114·66 | 126·63 | 86·04 | Antiretroviral drugs | 62·31 | 71·31 | 61·57 | 60·23 |
| Total (2018) | 129·31 | 160 | 185·34 | 99·83 | Total (2018) | 88·44 | 116·08 | 109·84 | 74·02 |
| inflated to 2021 | 153·62 | 190·08 | 220·18 | 118·60 | inflated to 2021 | 105·06 | 137·90 | 130·49 | 87·93 |
| **LMICs in Africa** | | | | | **LMICs in Africa** | | | | |
| Facility visit | 5·07 | 4·39 | 0·99 | 4·5 | Facility visit | 5·07 | 4·39 | 0·99 | 4·5 |
| Pharmacy pickup | 3·61 | 5·67 | 2·34 | 3·89 | Pharmacy pickup | 3·61 | 5·67 | 2·34 | 3·89 |
| DSD visits | 11 | 10·36 | 46·63 | 0 | DSD visits | 9·15 | 9·83 | 36.96 | 0 |
| Laboratory testing | 6·92 | 23·24 | 4·56 | 4·61 | Laboratory testing | 6·92 | 23·24 | 4.56 | 4·61 |
| Non-antiretroviral drugs | 0·1 | 0·18 | 0·18 | 0·13 | Non-antiretroviral drugs | 0·1 | 0·18 | 0.18 | 0·13 |
| Antiretroviral drugs | 101·21 | 114·66 | 126·63 | 86·04 | Antiretroviral drugs | 62·31 | 71·31 | 61.57 | 60·23 |
| Total (2018) | 127·91 | 158·50 | 181·33 | 99·17 | Total (2018) | 87·16 | 114·62 | 106.60 | 73·36 |
| inflated to 2021 | 148·19 | 183·63 | 210·08 | 114·89 | inflated to 2021 | 100·97 | 132·79 | 123·50 | 84·99 |

**Table S6. Point estimates of ICERs** for DSDs compared to SoC by countries and mathematical models in the main analysis (3% discounted).

| **($/DALYs averted)** | **Zambia** | **South Africa** | **Malawi** | **LMICs in Africa** |
| --- | --- | --- | --- | --- |
| **CAG** | 469 | 604 | 429 | 274 |
| **UAG** | 618 | 634 | 720 | 590 |
| **HomeART** | 841 | 1486 | 910 | 640 |

**Table S7. Country-specific income categories, GDP per capita, and health worker earning index for costs adjustment.**

| **Country** | **World Bank income category (2010)** | **GDP per capita (USD, 2010)** | **Earning Index^∇^** | | **Adjustment ratio** | |
| --- | --- | --- | --- | --- | --- | --- |
|  |  |  | **Nurses^∏^** | **Others^∏^** | **Nurses^∏^** | **Others^∏^** |
| **Zambia** | lower-middle- | 1469·4 | 4·2 | 2·4 | Ref (1·00) | |
| **South Africa** | upper-middle- | 8059·6 | 2·2 | 1·3 | 2·87 | 2·97 |
| **Malawi** | lower- | 472·9 | 6·4 | 3·7 | 0·49 | 0·50 |
| **LMICs in Africa** | lower-middle- | 1655·1 | 4·2 | 2·4 | 1·13 | 1·13 |

**^∇^** The earning index is a multiplier of GDP per capita.

**^∏^** ‘Others’ and ‘nurses’ represent the level of health worker cadre, classified by the level of education/training received, which is associated with their salaries. ‘Nurses’ is equivalent to health workers receiving the first stage of tertiary education, which is applicable to healthcare staff involved during facility visits. ‘Others’ is equivalent to health workers receiving below tertiary education, which is applicable to “DSD visits” and “pharmacy pickup”.

**Table S8. Point estimates for ICERs of DSDs under the alternative YLLs calculation approach** during the one-way sensitivity analysis (3% discounted).

| **($/DALYs averted)** | **Zambia** | **South Africa** | **Malawi** | **LMICs in Africa** |
| --- | --- | --- | --- | --- |
| **CAG** | 379 | 516 | 359 | - |
| **UAG** | 518 | 566 | 615 | - |
| **HomeART** | 623 | 1131 | 719 | - |

## **Supplementary Figures**


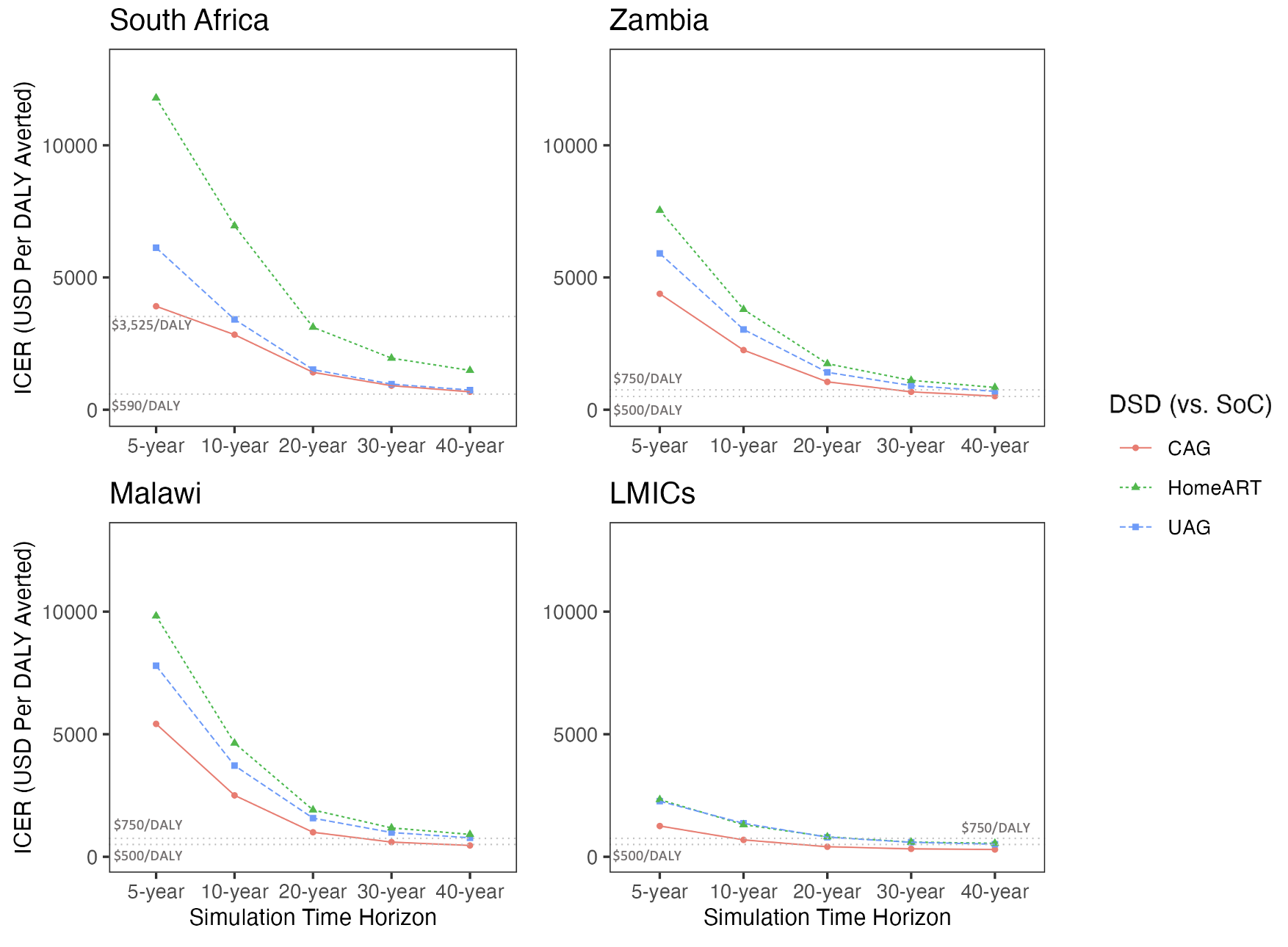


**Figure S1.** **Incremental cost-effectiveness ratios (ICERs) of DSD compared to SoC over different time horizons.** The dashed grey lines represented country-specific upper and lower CE thresholds. *Abbreviations: CAG: community adherence group; UAG: urban adherence group; HomeART: Community HIV epidemic control model; DSD: differentiated service delivery.*

**Alt text**: Line plots showing incremental cost-effectiveness ratio (USD per DALY averted) over 5-, 10-, 20-, 30-, and 40-year simulation horizons for South Africa, Zambia, Malawi, and LMICs. Lines are colored by differentiated service delivery implementation. ICERs decrease with longer time horizons across all countries. Community adherence group consistently yields the lowest ICERs compared with urban adherence group and home ART delivery. Grey dotted horizontal lines indicate country-specific cost-effectiveness thresholds.


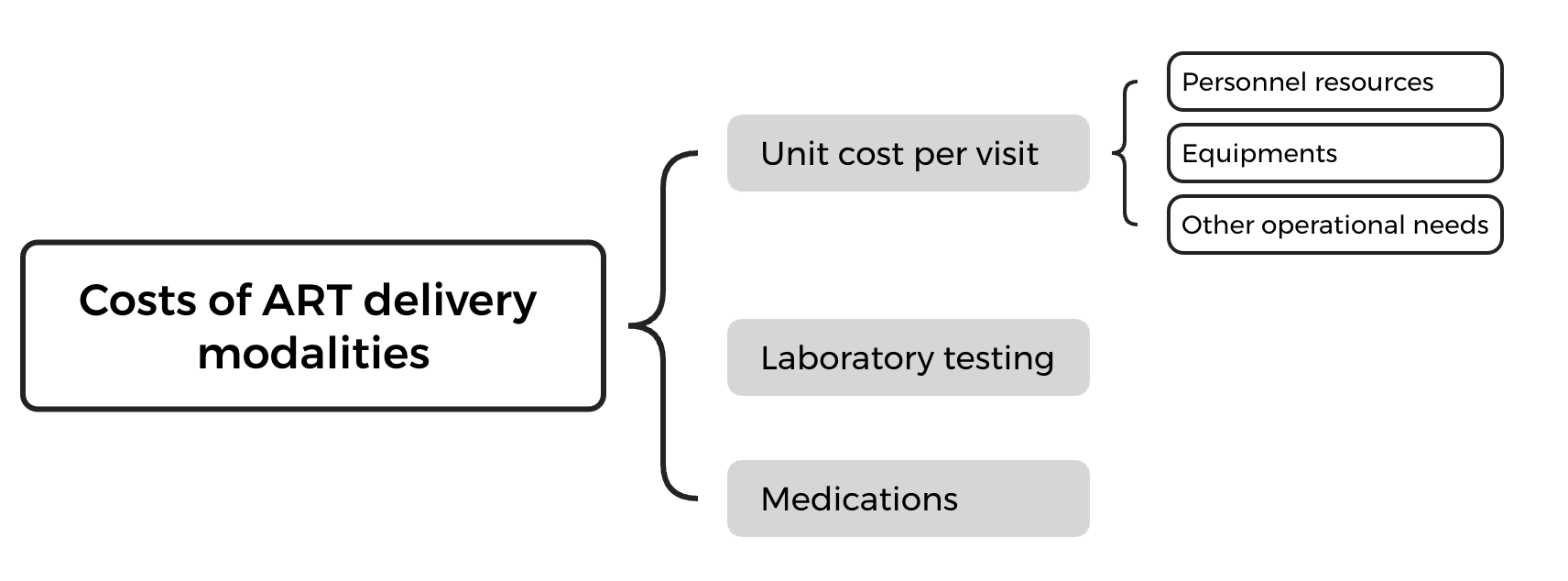


**Figure S2. Cost breakdowns for antiretroviral therapy delivery modalities.**

**Alt text**: Diagram illustrating cost components of antiretroviral delivery modalities. Costs include unit cost per visit – covering personnel resources, equipment, and other operational needs – along with laboratory testing and medications.

# **Supplementary References**

S1. Nichols BE, Cele R, Jamieson L, et al. Community-based delivery of HIV treatment in Zambia: costs and outcomes. *Aids* 2021; **35**(2): 299-306.

S2. Serje J, Bertram MY, Brindley C, Lauer JA. Global health worker salary estimates: an econometric analysis of global earnings data. *Cost Effectiveness and Resource Allocation* 2018; **16**: 1-9.

S3. The World Bank. GDP per capita by countries. 2023. https://data.worldbank.org/indicator/NY.GDP.PCAP.CD.

S4. The World Bank. The World by Income and Region. 2023. <https://datatopics.worldbank.org/world-development-indicators/the-world-by-income-and-region.html>.

S5. WHO G. WHO methods and data sources for global burden of disease estimates 2000–2011. *Geneva: Department of Health Statistics and Information Systems* 2013.

S6. Andrew Phillips, Loveleen Bansi-Matharu, Valentina Cambiano, Jenny Smith, Emily Dubrovska, McLeod M. HIV Synthesis. <http://hivmodeling.org/model-database/hiv-synthesis2024>).

S7. Bansi-Matharu L, Mudimu E, Martin-Hughes R, et al. Cost-effectiveness of voluntary medical male circumcision for HIV prevention across sub-Saharan Africa: results from five independent models. *Lancet Glob Health* 2023; **11**(2): e244-e55.
